# Integrative Bioinformatics Analysis Identifies DDX60 as a Potential Biomarker for Systemic Lupus Erythematosus

**DOI:** 10.1155/2023/8564650

**Published:** 2023-01-09

**Authors:** Wu Chen, Zhi-Yu Li, Lin Huang, Dong-Hai Zhou, Wen-Qing Luo, Xu-Feng Zhang, Lin Li, Cheng-Ping Wen, Qiao Wang

**Affiliations:** ^1^Zhejiang Chinese Medical University, Hangzhou, Zhejiang, China; ^2^The First Affiliated Hospital of Zhejiang Chinese Medical University (Zhejiang Provincial Hospital of Chinese Medicine), Hangzhou, China; ^3^Key Laboratory of Chinese Medicine Rheumatology of Zhejiang Province, China; ^4^The Second Affiliated Hospital of Zhejiang Chinese Medical University, Hangzhou, Zhejiang, China

## Abstract

**Background:**

Systemic lupus erythematosus (SLE) is an autoimmune disease with strong heterogeneity, leading to variable clinical symptoms, which makes diagnosis and activity evaluation difficult.

**Methods:**

The original dataset of GSE88884 was analyzed to screen differentially expressed genes (DEGs) of SLE and the correlation between DEGs and clinical parameters (SLEDAI, anti-dsDNA, C3, and C4). The result was validated by microarray GSE121239 and SLE patients with RT-qPCR. Next, receiver operator characteristic (ROC) analysis, correlation analysis, and ordinal logistic regression were applied, respectively, to evaluate the capability of diagnosis and prediction of the candidate biomarker. Subsequently, the biological functions of the candidate biomarker were investigated through KEGG and GO enrichment, protein–protein interaction network, and the correlation matrix.

**Results:**

A total of 283 DEGs were screened, and seven of them were overlapped with SLE-related genes. DDX60 was identified as the candidate biomarker. Analyses of GSE88884, GSE121239, and SLE patients with RT-qPCR indicated that DDX60 expression level is significantly higher in patients with high disease activity. ROC analysis and the area under the ROC curve (AUC = 0.8818) suggested that DDX60 has good diagnostic performance. DDX60 expression level was positively correlated with SLEDAI scores (*r* = 0.24). For every 1-unit increase in DDX60 expression value, the odds of a higher stage of activity of SLE disease are multiplied by 1.47. The function of DDX60 mainly focuses on IFN-I-induced antiviral activities, RIG-I signaling, and innate immune. Moreover, DDX60 plays a synergistic role with DDX58, IFIH1, OASL, IFIT1, and other related genes in the SLE pathogenesis*. Conclusions*. DDX60 is differently expressed in SLE, and it is significantly related to both serological indicators and the disease activity of SLE. We suggested that DDX60 might be a potential biomarker for SLE diagnosis and management.

## 1. Introduction

Systemic lupus erythematosus (SLE) is a chronic inflammatory autoimmune disease with variable clinical manifestations. It is characterized by repeated flares that seriously affect the quality of life. The heterogeneity of SLE brings a challenge for its diagnosis and management [1]. However, accurate monitoring of disease activity is critical for the management of SLE.

Serologic indicators, including anti-double-stranded DNA (anti-dsDNA), complement component C3 (C3), and complement component C4 (C4), are essential in diagnosing and assessment of disease activity. However, a large single-center cohort study revealed that fluctuations in anti-dsDNA or complement levels are not sensitive in predicting flares [2]. It is also widely believed that serological parameters are not positively related with clinical symptoms such as joint pain. Therefore, a composite biomarker is needed that can help diagnose SLE and monitor the state of the disease.

Type I interferons (IFNs) have been reported to be a major pathogenic factor in SLE [3]. Type I IFNs induce DExD/H-Box helicase 60 (DDX60) as demonstrated by viral infection-induced gene microarray analysis in human dendritic cells [4]; DDX60 is an uncharacterized DEXD/H-box RNA helicase, similar to *S. cerevisiae* Ski2, a protein complex required for cytoplasmic RNA integrity. In innate immune cells, DDX46 can demethylate m~6A-modified antiviral gene mRNA through its helicase domain binding ALKBH5 and then retain it in the nucleus during viral infection, further inhibit interferon production, and inhibit antiviral innate immunity. Therefore, this theory may be involved in the pathophysiology of SLE. Increased DDX60 expression in CD14 monocytes has been reported in childhood-onset SLE patients; this effect is thought to be characteristic of type I IFN activation [5]. However, the role of DDX60 in SLE has not been reported. In this study, DDX60 was identified as a potential biomarker of SLE. We found that the expression level of DDX60 was related to SLEDAI as well as the anti-dsDNA, C3, and C4. ROC (receiver operator characteristic) analysis and correlation analysis indicate that DDX60 has good performance for SLE diagnosis and disease activity prediction. Our findings suggest that DDX60 reflects both the immune inflammation level and the overall clinical activity of SLE and might be a potential biomarker for better diagnosis and management of SLE.

**Figure 1:**
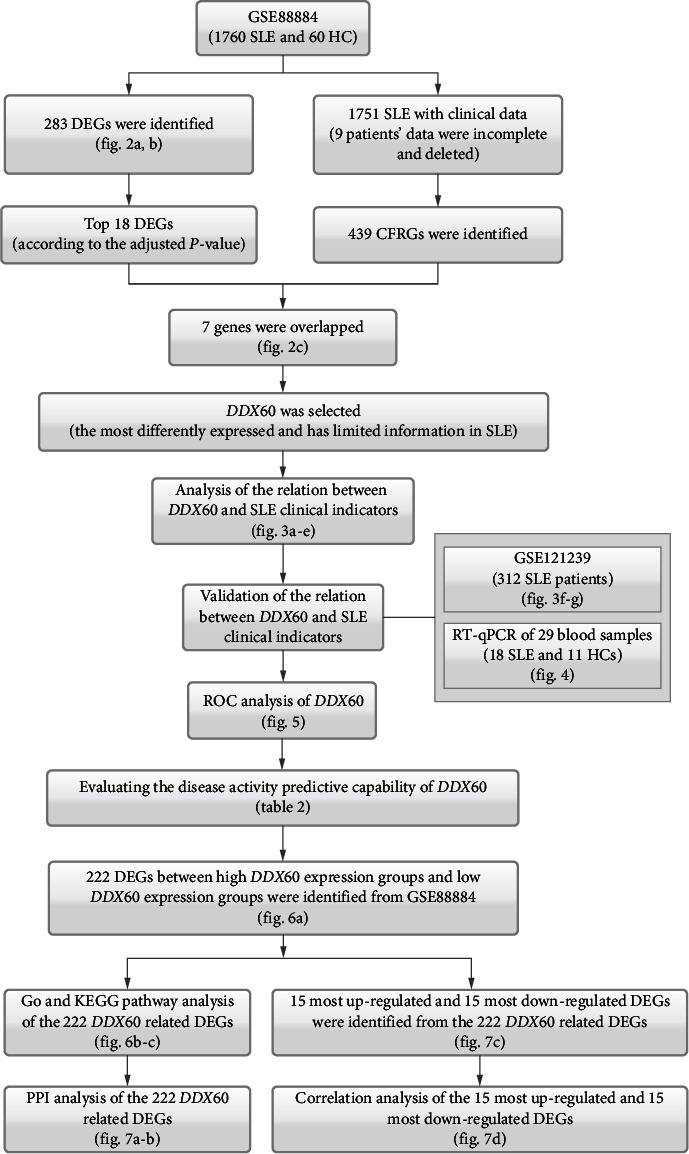
The flow chart of the study. SLE: systemic lupus erythematosus; HCs: healthy controls; DEGs: differentially expressed genes; CFRGs: clinical feature-related genes; DDX60: DExD/H-Box helicase 60; ROC: the receiver operator characteristic (ROC) curve; GO: Gene Ontology; KEGG: Kyoto Encyclopedia of Genes and Genomes.

The flow chart of the study is shown in Figure 1.

## 2. Materials and Methods

### 2.1. Identification of Differentially Expressed Genes

GSE88884 was obtained from the GEO database (https://www.ncbi.nlm.nih.gov/gds/). It contains the baseline gene expression profiling data as well as the clinical and laboratory characteristics of 1760 SLE patients and 60 healthy controls (HCs) from two phase III, 52 week, randomized, placebo-controlled, double-blind, parallel studies (Table S1). Patients meet with 1997 ACR classification criteria were diagnosed as SLE [6]. Detailed inclusion and exclusion criteria are shown in Information S1. In order to get a list of the relationship between probes of the chip corresponding to gene IDs, gene probes were transferred to gene symbols according to the platform file—GPL17586. The “limma” package is a tool for performing differential analysis. The probe with the maximum expression was selected if there were duplicate probe names. Then, the differentially expressed genes (DEGs) between SLE patients and HCs were screened by the “limma” package with the criteria of a ^“^log fold change^”^ > 0.5 and an adjusted *P* value < 0.01. The heatmap and volcano map of the DEGs were visualized by the R software.

### 2.2. Identification of Clinical Feature-Related DEGs

The clinical data of 1751 SLE patients (9 were incomplete and deleted, listed in Table S1) from GSE88884, including SLEDAI scores, anti-dsDNA, C3 level, and C4 level, were arranged. Patients were classified into different groups according to clinical assessments, as shown in Table 1.

Genes differentially expressed in any two subgroups of any of the four clinical evaluations were identified as SLE clinical feature-related genes (CFRG) by R software. Intergroup differences were evaluated by the Wilcox test and the Kruskal-Wallis test with a *P* value < 0.01. The DEG and CFRG overlap genes were operated by the FunRich software (version 3.1.3).

### 2.3. Analysis of the Relation between the Candidate Gene and Clinical Data

The candidate gene was classed into four subgroups according to the four clinical indicators SLEDAI scores, anti-dsDNA, C3, and C4. The relationship of the candidate gene and four clinical evaluations was analyzed and visualized by the “ggpubr” software package. Intergroup differences were evaluated by the Kruskal-Wallis test.

### 2.4. Confirming the Results Using Another Microarray GSE121239

To further verify the role of DDX60 in SLE, GSE121239 microarray (312 samples) was downloaded from the GEO database, which contains longitudinal disease activity and whole-genome gene expression data of 20 HCs and 65 SLE patients with more than three visits. SLE was defined according to the 1997 ACR classification criteria or the Systemic Lupus International Collaborating Clinics classification criteria [6, 7]. The 92% of the patients were female with a median age of 47 years (interquartile range 37–55). Patients are consisted of 58% Caucasian American, 35% African American, and 7% of other race/ethnic groups. The average SELENA-SLEDAI at baseline was 2.4 (SD = 2.6), ranging from 0 to 12. Of the 243 patients in the analysis, 62 had SLEDAI score of 0 for all follow-up visits. The microarray analysis method of GSE121239 is mentioned above.

### 2.5. RT-qPCR Validation of the Relation between the Candidate Gene Expression and Clinical Data

To validate the expression of the candidate gene in patients with SLE and HCs, whole blood transcriptional expression of the candidate gene was analyzed by RT-qPCR. Outpatients who met with 1997 ACR classification criteria and has normal white blood cell and lymphocyte count and HCs were recruited from the Second Affiliated Hospital of Zhejiang Chinese Medical University during October to November in 2020 [6]. The detail information of the subjects was given in Table S3. The study was reviewed and approved by the Institutional Review Board of the Zhejiang Chinese Medical University. All participants signed the informed consent.

A total of 1 mL of fresh whole blood samples were collected from each participant, and total RNA was extracted using TRIzol reagent (Takara, Kusatsu, Shiga, Japan). Total RNA was reversed to cDNA using ReverTra Ace qPCR RT Kit (Toyobo, Osaka, Japan) in a T100TM Thermal Cycler (Bio-Rad, CA, USA). The cDNA was then subjected to RT-qPCR using UltraSYBR Mixture (cwbiotech, Taizhou, Jiangsu, China) in a Light Cycler 96 (Roche, Basel, Switzerland). The primer sequences were designed according to an online website. The following qPCR primers were used: *DDX60*, forward, 5′-CAGCTCCAATGAAATGGTGCC-3′, and reverse, 5′-CTCAGGGGTTTATGAGAATGCC′; GAPDH, forward, 5′-TCAAGGCTGAGAACGGGAAG-3′, and reverse, 5′-GACTCCACGACGTACTCAGC′. RT-qPCR data were presented as mean with 95% confidence interval (CI). Outliers were identified using Grubb's method. The significance of the difference between groups was determined by unpaired *t*-test (Student's *t*-test or Welch's *t*-test) after determining the variances using the *F* test. Differences with *P* < 0.05 were considered statistically significant. All statistics were analyzed and visualized with GraphPad Prism 9.1.1 (GraphPad Software, San Diego, USA).

### 2.6. Receiver Operator Characteristic Analysis of the Biomarker

We used simple logistic regression to fit a model to predict the presence of SLE based on the expression value of the candidate biomarker. The biomarker expression data of SLE patients and HCs were collected from GSE88884. Then, the ROC analysis was performed to assess the diagnostic performance of the biomarker, including the calculation of the area under the ROC curve (AUC). An AUC > 0.8 indicates that the predicted model has good efficacy. Wald's test and likelihood ratio test were used to test if the odds ratio (OR) is 1.0. Outliers were identified using the ROUT method (*Q* = 1%). All statistical analyses were performed with GraphPad Prism 9.1.1 (GraphPad Software, San Diego, USA).

### 2.7. Evaluating the Predictive Capacity of Disease Activity of the Biomarker

Correlation analysis and ordinal logistic regression were conducted to evaluate the relation between candidate biomarker expression and SLE disease activity with GraphPad Prism 9.1.1. Correlation analysis was performed using Spearman's rank correlation test, and the D'Agostino and Pearson omnibus normality test was used to test for normal distribution. The candidate biomarker expression data and SLEDAI scores were collected from GSE88884. Then, the ordinal logistic regression was conducted using SPSS 26.0.0 (IBM SPSS Statistics, IBM, USA). The expression value of the candidate biomarker was used as the independent variable, and SLE disease activity was the dependent variable. As an ordinal variable, SLE disease activity was classified into four stages according to SLEDAI scores from low to high: 0-4, inactive; 5-9, mild active; 10-14, moderate active; and ≥15, severe active. Test of parallel lines was performed before the ordinal logistic regression. A *P* value ≥ 0.05 indicates that the location parameters (slope coefficients) are the same across response categories.

### 2.8. Identification of Candidate Biomarker-Related DEGs

According to the median expression level of *DDX60*, 1820 samples were divided into “high expression group” and “low expression group.” DEGs between the high expression group and the low expression group were screened by the package “limma” with a ^“^log fold change^”^ > 0.5 and an adjusted *P* value < 0.01. The volcano plot was performed with the R software.

### 2.9. Gene Ontology and KEGG Pathway Analyses of Candidate Biomarker-Related Genes

The “org.Hs.eg.db” package mainly annotates human genes for transformation between different database IDs. Gene symbols were converted into gene ID using the “org.hs.eg/.db” package. Gene Ontology (GO) and Kyoto Encyclopedia of Genes and Genomes (KEGG) analyses were performed by the “clusterProfiler” and “enrichplot” packages in the R program.

### 2.10. Protein–Protein Interaction Network Construction and Correlation Analysis of Candidate Biomarker-Related DEGs

The protein–protein interaction (PPI) network data of the DEGs between the high *DDX60* expression group and low *DDX60* expression group was derived from the Search Tool for the Retrieval of Interacting Genes database (https://string-db.org/). Cytoscape (version 3.5.0) was applied to construct and visualize the PPI network (https://cytoscape.org/). The Molecular Complex Detection (MCODE) plug-in (version 2.0) of Cytoscape was employed to identify highly connected subclusters with the degree cutoff = 2, max. depth = 30, *k*‐core = 2, and node score cutoff = 0.1. The heatmap of the 15 most significantly upregulated and 15 most downregulated DEGs was performed by R. The correlation matrix was visualized by the “corrplot package” in R.

## 3. Results

### 3.1. Identification of the Candidate Biomarker

**Figure 2:**
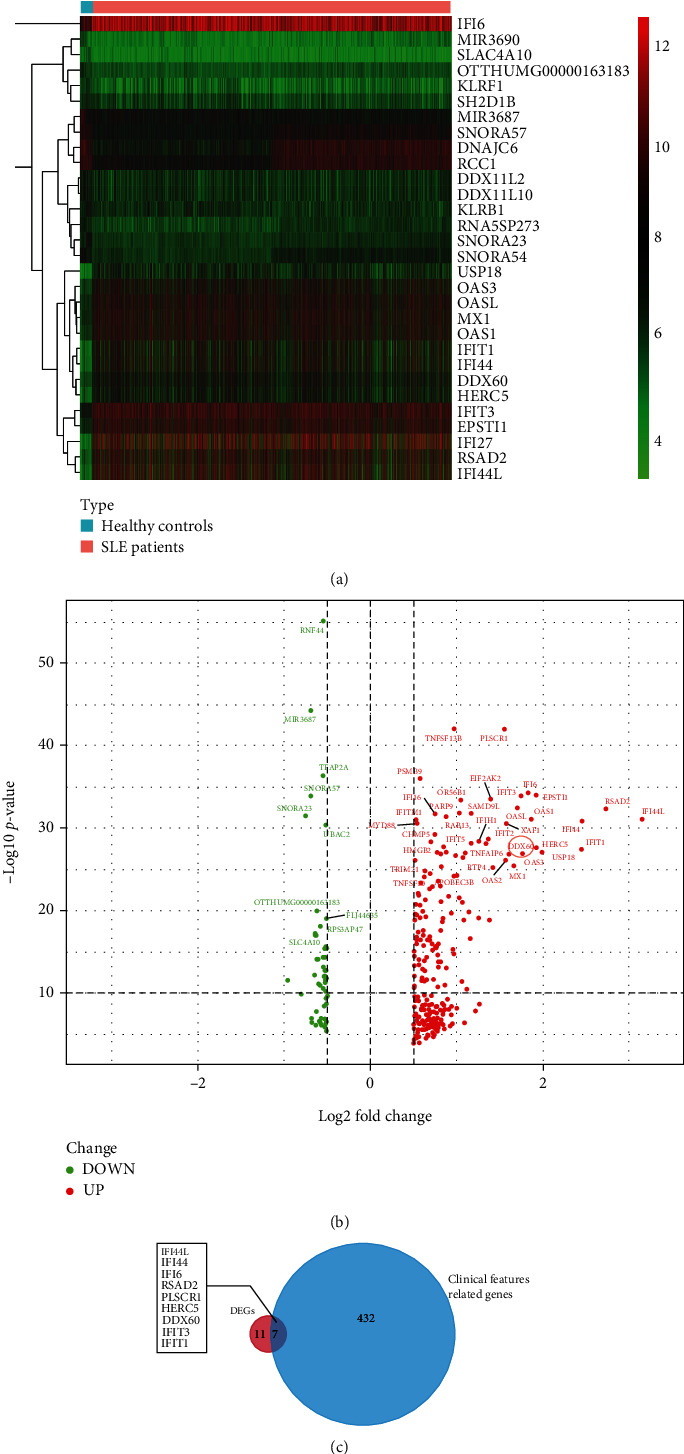
Differentially expressed gene (DEG) expression profile between healthy controls and SLE patients from GSE88884. (a) Heatmap indicates the top 15 upregulated (red) and downregulated (green) DEGs. (b) Volcano plot showed 231 upregulated (red) and 52 downregulated (green) DEGs. (c) The Venn chart showed the seven overlapped genes of the 18 most differently expressed DEGs and 439 clinical feature-related genes from GSE88884.

We identified 283 genes as DEGs (Figures 2(a) and 2(b)) in SLE patients as compared with HCs, including 231 upregulated and 52 downregulated genes. A total of 439 genes were identified as CFRGs. Seven DEGs, namely, DDX60, IFI44L, IFI6, IFI44, RSAD2, PLSCR1, and HERC5, overlapped with 439 CFRGs (Figure 2(c)). Most of these seven genes have been reported to be related to SLE (Table S4). However, the role of DDX60 in SLE pathogenesis remains unclear.

### 3.2. Relative Expression Levels of DDX60 in Different Groups of Clinical Evaluation Indicators

**Figure 3:**
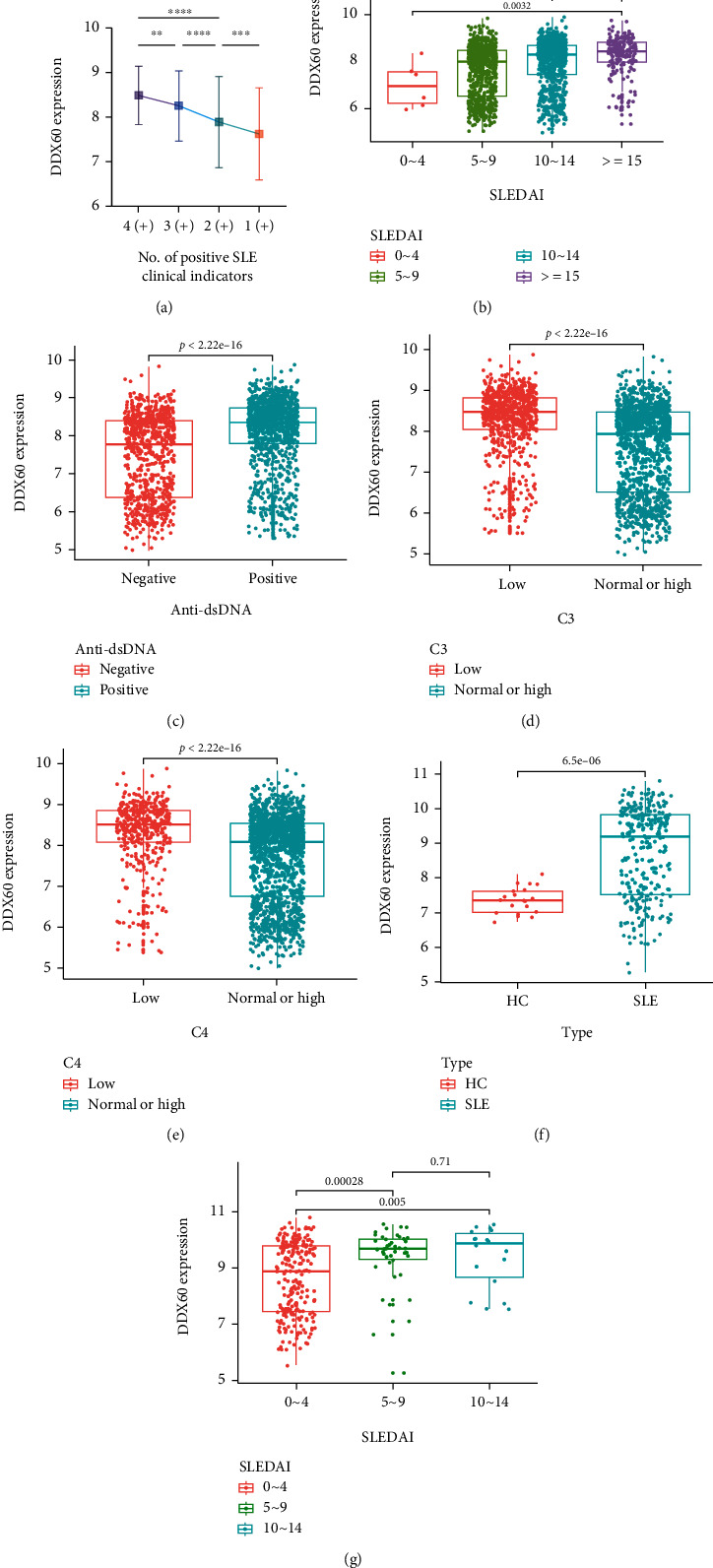
*DDX60* expression levels of different subgroups according to SLE Disease Activity Index (SLEDAI), anti-dsDNA, complement component C3 (C3) level, and complement component C4 (C4) level in GSE88884 and GSE121239. (a) *DDX60* expression levels of the four subgroups that were distinguished according to the number of positive indicators (SLEDAI ≥ mild active, anti-dsDNA = “positive,” C3 = “low,” and C4 = “low”). ^∗∗∗∗^*P* < 0.0001; ^∗∗∗^*P* < 0.001; ^∗∗^*P* < 0.01; ^∗^*P* < 0.05; error bars show the standard deviation of the mean. (b) *DDX60* levels in patients with inactive SLE (0 ~ 4), mild active (5 ~ 9), moderate active (10~ 14), and severe active SLE (≥15) of GSE88884. (c) *DDX60* levels in the negative or positive anti-dsDNA subgroups of GSE88884. (d, e) DDX60 levels in patients with low C3/C4 level and normal or high level from GSE88884. (f) DDX60 expression level in systemic lupus erythematosus (SLE) patients is significantly higher than in healthy controls (HC) from GSE121239 (*P* < 0.0001). (g) The expression levels of DDX60 of patients with the SLEDAI scores 10-14 and 5-9 are significantly higher than those of score 0-4 (*P* < 0.05) from GSE121239. Box-whisker plots show median, interquartile range, nonoutlier range, and outliers.

The relative expression levels of DDX60 in 1751 SLE patients were analyzed. As the number of positive indicators (SLEDAI ≥ mild active, anti-dsDNA = “positive,” C3 = “low,” and C4 = “low” according to the classification in Table 1) in the four indicators increased, the DDX60 expression level increased significantly accordingly (Figure 3(a)). Furthermore, the expression levels of DDX60 differed among the four SLEDAI groups, except for the no-activity group from the mild-activity group, with a positive relation trend between DDX60 expression levels and SLEDAI scores (Figure 3(b)). As shown in Figure 3(c), the DDX60 expression level in anti-dsDNA positive patients was significantly higher than in anti-dsDNA negative patients (*P* < 0.0001). Moreover, the expression levels of DDX60 were significantly higher in patients with low C3 and C4 levels than in patients with normal or high levels (*P* < 0.0001) (Figures 3(d) and 3(e)).

### 3.3. Validation of the Relation between DDX60 and Clinical Features of SLE

The *DDX60* expression level of SLE patients is significantly higher than that of HCs (*P* < 0.0001) in microarray GSE121239 (Figure 3(f)). As shown in Figure 3(g), DDX60 expression levels of mild and moderate active patients are significantly higher than inactive patients (*P* < 0.05).

**Figure 4:**
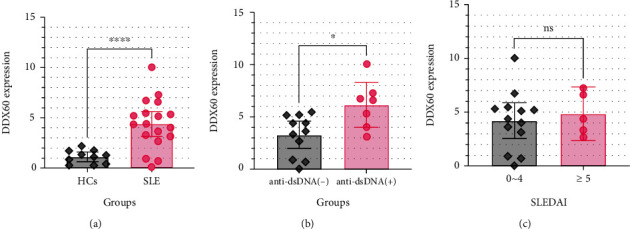
DDX60 expression levels in different subgroups of the SLE Disease Activity Index (SLEDAI) and anti-dsDNA in blood samples. ^∗∗∗∗^*P* < 0.0001; ^∗^*P* < 0.05; ns: no statistically significant difference (*P* ≥ 0.05). (a) DDX60 expression level of patients with systemic lupus erythematosus (SLE) was significantly higher than that of healthy controls (HCs) (*P* < 0.0001). (b) DDX60 expression level of patients with positive anti-dsDNA antibody was higher than that of patients with negative dsDNA antibody. (c) No significant difference (*P* > 0.05) of DDX60 expression level was observed between patients with SLEDAI ≥ 5 and patients with SLEDAI from 0 to 4.

Then, we performed RT-qPCR on clinical samples to validate the speculation of the relation between DDX60 expression level and SLE clinical evaluations. A total of 29 peripheral blood samples and related clinical information were obtained from 18 female SLE patients and 11 female HCs. The DDX60 expression value of one HC (3.832) was identified as an outlier and excluded. Patients' specific information was listed in Table S4. As shown in Figure 4(a), the DDX60 expression level of SLE patients was significantly higher than that of healthy controls (HCs) (*P* < 0.0001). Furthermore, the level of DDX60 expression of patients with positive anti-dsDNA antibody was higher than that of patients with negative dsDNA antibody (Figure 4(b)). However, no significant difference (*P* > 0.05) in DDX60 expression level was observed between patients with SLEDAI ≥ 5 and patients with SLEDAI from 0 to 4 (Figure 4(c)).

### 3.4. Receiver Operator Characteristic Analysis of DDX60

**Figure 5:**
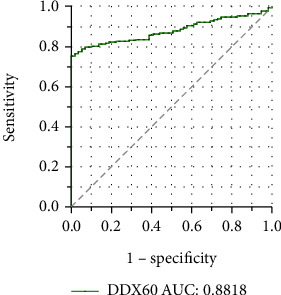
The receiver operator characteristic (ROC) curve of DDX60. The curve represents the diagnostic performance of DDX60 in SLE patients. The AUC is 0.8818 (95% CI 0.8614 to 0.9021, *P* < 0.0001). An AUC > 0.8 indicates that the predicted model has good efficacy. ROC: receiver operator characteristic; AUC: area under the ROC curve.

The result of simple logistic regression was shown in Table S5, indicating that DDX60 expression has a significant effect on the diagnosis of SLE (OR 4.608 (95% CI 3.328 to 6.706), *P* < 0.0001). The ROC curve is presented in Figure 5, with an AUC of 0.8818 (95% CI 0.8614 to 0.9021, *P* < 0.0001), suggesting that DDX60 has a good diagnostic performance. One DDX60 expression value (8.304) from HCs was identified as an outlier and excluded.

### 3.5. The Predictive Capacity of Disease Activity of DDX60

Spearman's rank correlation test showed a positive correlation (*r* = 0.24 (95% CI: 0.19 to 0.28), *P* < 0.0001) between DDX60 expression and SLEDAI scores (Table S6). Moreover, the results of ordinal logistic regression for DDX60 expression and SLEDAI indicate that DDX60 expression has a definite effect on SLE disease activity (Table 2). For every 1-unit increase in the expression value of DDX60, the odds of a higher stage of SLE disease activity are multiplied by 1.47 (95% CI (1.349-1.611), *P* < 0.0001).

### 3.6. KEGG and GO Pathway Analyses of DDX60-Related Genes

**Figure 6:**
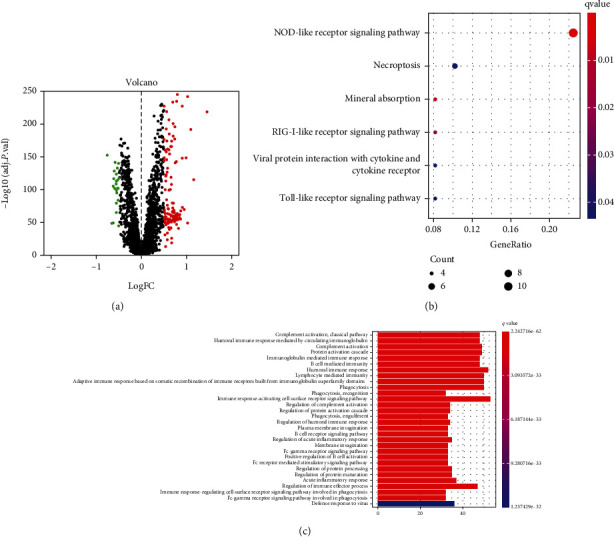
Volcano plot and the Kyoto Encyclopedia of Genes and Genomes (KEGG) and Gene Ontology (GO) enrichment analyses of DDX60-related DEGs. (a) Volcano plot showed the 199 upregulated (red) and 23 downregulated (green) genes. (b) Bubble diagram illustrates the top significantly enriched pathways of KEGG analysis with an adj *P* value < 0.05. The size of the nodes indicates the number of enriched genes, and the colors represent the significant levels of enrichment. (c) Bar plot illustrates the top 30 significantly enriched GO terms. The length of the bar indicates the number of enriched genes in this GO term, and the colors represent the significance levels of enrichment.

A total of 222 DEGs (199 upregulated and 23 downregulated) were differentially expressed between the DDX60 high expression group and the low expression group (Figure 6(a)). To further investigate the biological functions of DDX60-related genes, the KEGG and GO analyses of 222 DEGs were performed. The KEGG pathway analysis displayed that the DEGs were mainly enriched in 6 pathways, including NOD-like receptor signaling pathway, mineral absorption, RIG-I-like receptor (RLR) signaling pathway, necroptosis, viral protein interaction with cytokine and cytokine receptor, and Toll-like receptor signaling pathway (Figure 6(b)). Besides, GO enrichment analysis indicated that DEGs were mainly enriched in 30 pathways, including complement activation, humoral immune response mediated by circulating immunoglobulin, protein activation cascade, and immunoglobulin- (Ig-) mediated immune response (Figure 6(c)).

### 3.7. PPI Network Construction and Correlation Analysis of DDX60-Related Genes

**Figure 7:**
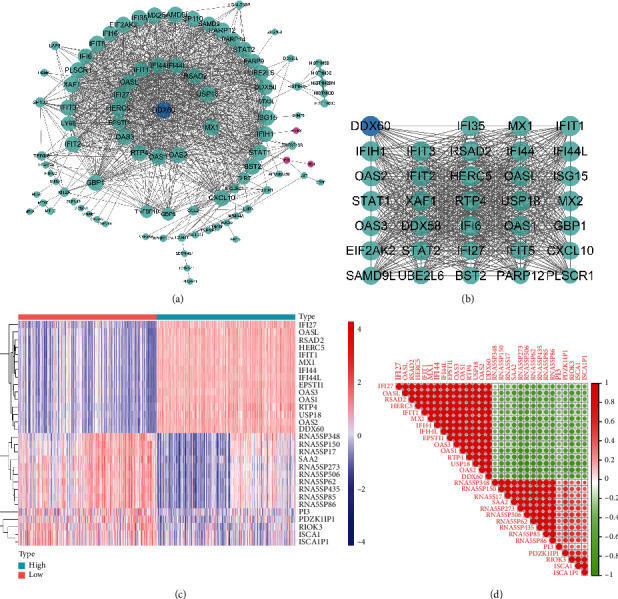
PPI network, heatmap, and correlation matrix of DDX60-related DEGs. (a) PPI network constructed with the DDX60-related DEGs. The green nodes represent upregulated genes, the pink nodes stand for downregulated genes, and the blue node is DDX60. The gradual change of node size from small to big indicates the MCODE score from small to large. (b) The most highly interconnected subcluster separated from the PPI network by the Molecular Complex Detection (MCODE) plug-in according to the MCODE score. The gradual change of node size from small to big indicates the degree value from small to large. (c) Heatmap of the 15 most significantly upregulated (red) and 15 most downregulated (blue) DDX60-related DEGs. (d) Correlation matrix shows the intercorrelation of the 15 most upregulated and 15 downregulated genes. The colors refer to the correlation coefficient magnitude, ranging from positive (red) to negative (green) correlation. Large circles indicate strong correlations, whereas small ones reflect weak correlations.

To further explore the interaction between DDX60 and its 222 related DEGs, a PPI network consisting of 95 nodes and 956 edges was constructed (Figure 7(a)). The most highly interconnected subcluster was separated from the PPI network by the MCODE plug-in according to the MCODE score (Figure 7(b)). All nodes in this subcluster were connected to DDX60, indicating that DDX60 was one of the hub genes of the subcluster.

Moreover, the 15 most upregulated and 15 most downregulated DEGs of the 222 DDX60-related DEGs are shown in the heatmap (Figure 7(c)). Then, the correlation of these 30 DEGs was analyzed and visualized by the correlation matrix (Figure 7(d)).

## 4. Discussion

In this study, we applied bioinformatics to search for genes that are both related to SLE clinical evaluation scales (SLEDAI) and serological indicators (anti-dsDNA, C3, and C4) and discovered DDX60 as the candidate biomarker. We found that DDX60 expression level is positively related with disease activity and serological indicators' level. Then, a second microarray, GSE121239, and RT-qPCR performed on peripheral blood samples of SLE patients, and HCs were used to verify the findings. Furthermore, simple logistic regression and ROC analysis indicated that DDX60 has a significant effect on SLE diagnosis and might have good diagnostic performance. Moreover, the results of ordinal logistic regression for DDX60 expression and SLEDAI suggested that DDX60 might have a good predictive capacity of SLE disease activity.

DDX60 codes an antiviral protein, a novel DEAD-box RNA helicase. However, the role of DDX60 in SLE has not been reported. To investigate the biological function of DDX60 in SLE, we first identified 222 DEGs that were differentially expressed between the DDX60 high expression and DDX60 low expression groups. Then, we performed KEGG and GO pathway enrichment for these 222 *DDX60-*related DEGs. KEGG analysis showed that DDX60-related DEGs were enriched mainly in 6 pathways (Figure 6(b)), while GO analysis showed that DEGs were enriched in 30 pathways (Figure 6(c)). Most of these pathways were involved in antiviral immune response, innate immune, type I IFN (interferon) induction, RLR signaling, complement activation, and immune regulation and were confirmed to have a vital role in SLE. For example, virus infection is one of the environmental factors of SLE, and the antiviral immune response plays a central role in the pathogenesis of SLE [8–10]. Viral RNA or DNA stimulates the production of large amounts of autoantibodies through molecular simulation mechanisms. These autoantigens further expand epitopes and react with other autoantigens, leading to activation of the immune system and promoting the development of SLE [11, 12]. The nucleic acid of the virus can be recognized by RLRs, which induces the production of IFN and leads to the expression of hundreds of interferon-stimulated genes (ISGs). ISGs mediate various immune cell effects similar to antiviral immune responses. The expression of ISGs was significantly higher in patients with SLE than in HC [13–16]. The widespread activation of ISGs and their regulation of the immune system have been demonstrated to be central mechanisms in SLE pathogenesis [17, 18]. Immune complex (IC) formed by autoantibodies in SLE patients with IFN-I signature will further induce the production of IFN-I [19]. The IFN-I system is continuously activated and drives autoimmune responses and chronic inflammation leading to multiple tissue damage in SLE.

DDX60 played an important role in these biological mechanisms. DDX60 is highly expressed in various viral infections and autoimmune diseases [20–24]. DDX60 is induced by virus RNA or IFN and functions as a sentinel for the cytoplasmic antiviral innate immune response [24]. DDX60 localizes and binds viral RNA, activates RIG-I signaling, and promotes RIG-I recognition and binding of viral dsRNA [4, 24, 25]. DDX60 positively regulates RIG-I-dependent IFN-I and ISG expression [4, 24, 26]. DDX60 knockout is described to weaken the RIG-I signal, reduce the IFN level, and increase the titer of the virus [26]. DDX60 and RIG-I induce the production of IFN-I, and IFN-I induces the expression of both DDX60 and DDX58, forming a circulation that leads to the continuous activation of the immune system and IFN system in SLE. SLE patients with high IFN-*α* expression have higher anti-dsDNA, ESR (erythrocyte sedimentation rate) levels, and lower complement levels [13], which may explain the relevance between DDX60 expression level and SLE activity indicators like C3, C4, anti-dsDNA, and SLEDAI.

Complement, one of the most commonly used clinical indices of SLE, is also the primary humoral medium of the innate immune response. As an adjuvant, complement plays a vital role in scavenging the IC formed by Ig binding with the antigen. Virus infection stimulates DDX60 expression and promotes RLR-dependent innate immune responses, accompanied by the production of large amounts of autoantibodies, antigen-antibody reactions, and the generation of ICs. Since the complement system plays a vital role in the clearance of immune complexes and the innate immune response, complement can be consumed in large amounts, resulting in reduced complement levels in SLE.

To further explore the role of DDX60 and its related genes in SLE, we firstly performed the PPI network analysis. Genes in the most highly interconnected subcluster (Figure 7(b)) were mostly ISGs and were all connected with DDX60 in the PPI network which suggests that these genes have a strong synergistic effect with DDX60 and together play a role in the IFN-related pathogenesis of SLE. For example, DDX58 encodes RIG-I, and IFIH1 encodes MDA5. RIG-I and MDA5 were two main RLRs, which were essential in the innate immune response to recognize viral RNA and induce IFN-I and play a central role in the pathogenesis of SLE [27]. Studies have reported the high expression level of RIG-I mRNA in urine sediment from patients with LN [28]. Upregulation of the expressions of RLR-related ISGs such as DDX58 and IFIH1 was found in childhood-onset SLE patients [5]. IFIH1 has been reported to be associated with SLE phenotype, anti-dsDNA, and susceptibility [29–31]. These RLRs are both ISGs and, therefore, are rapidly activated by IFN stimulation. RIG-I- and MDA5-dependent IFN-I expression and ISG expression were positively regulated by DDX60, as mentioned above.

Then, the correlation analysis of DDX60 and the 15 most upregulated DEGs along with the 15 most downregulated DEGs were analyzed (Figures 7(c) and 7(d)). The result shows that DDX60 and the 15 most upregulated DEGs were strongly correlated with each other, suggesting that DDX60 may have a strong synergistic effect with these genes. In addition, these 15 genes are consistent with those in the subcluster of the PPI network (Figure 7(b)). Most of these genes are IFN-I inducible and have been reported closely related to the disease activity, pathogenesis, and IFN signature expression of SLE [19, 32–36]. For example, upregulated genes OASL, OAS1, OAS2, and OAS3 belong to the OAS family, a group of antiviral enzymes induced by type I IFNs, which can locate and bind viral dsRNA after viral infection. In particular, OASL binds to RIG-I and activates and enhances RIG-I signaling; OAS1 binds to viral dsRNA and, together with DDX60, helps RIG-I recognize viral RNA and amplify viral-induced RIG-I-mediated type I IFN expression after viral infection [37–39]. Moreover, OAS1, OAS2, and OAS3 were closely related to lupus nephritis (LN) progression [36]. IFIT1, also induced by IFN stimulation and viral infection, plays an essential role in SLE immune disorders and tissue damage [14]. Knockdown of DDX60 was found to decrease IFIT1 expression [24]. In summary, the function of DDX60 and related genes mainly focused on antiviral immune responses, RIG-I signaling pathway, and type I IFN-mediated innate immune responses. These mechanisms also play a vital role in SLE and lead to its hyperimmune, autoimmunity, and chronic inflammation.

There are some limitations in this study. On the one hand, since there is no gold diagnostic standard for SLE, we were unable to verify its diagnostic ability with relevant data further. On the other hand, more studies are still needed on the predictive capacity of disease activity in the time dimension.

## 5. Conclusions

In this study, we identified that DDX60 was differently expressed in SLE patients. It was significantly related to both serological indicators and the overall clinical activity of SLE. We provided that high levels of DDX60 predicted high disease activity in SLE. Integrated bioinformatics analysis indicates that DDX60 may play a synergistic effect with its related genes in the signaling pathways of antiviral activity, RIG-I signaling pathway, and IFN-I-induced immune responses in SLE. The close association of DDX60 with immune inflammation and tissue damage in SLE may explain its association with both serological indicators and clinical scores of SLE. Collectively, our findings implied the potential clinical value of DDX60 in the diagnosis and management of SLE.

## Figures and Tables

**Table 1:** Classification of SLE patients according to four clinical assessments.

Clinical assessments	Classification
SLEDAI	0-4, inactive; 5-9, mild activity; 10-14, moderate activity; ≥15, severe activity
Anti-dsDNA	<30 IU was defined as the “negative” group and ≥30 IU as the “positive” group
C3	<0.9 g/L was defined as the “low” group and ≥0.9 g/L as the “normal or high” group
C4	<0.1 g/L was defined as the “low” group and ≥0.1 g/L as the “normal or high” group

SLEDAI: systemic lupus erythematosus disease activity index; anti-dsDNA: anti-double-stranded DNA; C3: complement component C3; C4: complement component C4.

**Table 2:** Ordinal logistic regression for DDX60 and SLEDAI.

Test of parallel lines		Model fitting		Ordinal logistic regression			
Chi-square	Sig.	Chi-square	Sig.	*B* (95% Wald's CI)	Wald's chi-square	Sig.	OR (95% Wald's CI)
2.361	0.307	76.649	<0.0001	0.388 (0.299 to 0.477)	73.336	<0.0001	1.474 (1.349 to 1.611)

CI: confidence interval; OR: odds ratio.

## Data Availability

The datasets GSE88884 and GSE121239 analyzed during the current study are available in the Gene Expression Omnibus. The websites of two datasets are listed as follows: https://www.ncbi.nlm.nih.gov/geo/query/acc.cgi?acc=GSE88884 and https://www.ncbi.nlm.nih.gov/geo/query/acc.cgi?acc=GSE121239.

## Abbreviations

SLE: Systemic lupus erythematosus
Anti-dsDNA: Anti-double-stranded DNA
C3: Complement component C3
C4: Complement component C4
SLEDAI: SLE Disease Activity Index
ROC: Receiver operator characteristic
HC: Healthy control
DEGs: Differentially expressed genes
CFRG: Clinical feature-related genes
CI: Confidence interval
AUC: Area under the ROC curve
OR: Odds ratio
GO: Gene Ontology
KEGG: Kyoto Encyclopedia of Genes and Genomes
PPI: Protein–protein interaction
MCODE: Molecular Complex Detection
Ig: Immunoglobulin
RLRs: RIG-I-like receptors
ISGs: Interferon-stimulated genes
IC: Immune complex
IFN: Interferon
ESR: Erythrocyte sedimentation rate.

## Supplementary Materials

Supplementary Materials Table S1: clinical information from GSE88884. Table S2: details of incomplete data of nine patients. Table S3: clinical information of 29 participants. Table S4: seven overlapped genes of DEGs and clinical feature-related genes from GSE88884. Table S5: simple logit regression of DDX60 expression in SLE/HC. Table S6: correlation analysis of DDX60 expression and SLEDAI in SLE patients. Information S1: patient inclusion criteria and exclusion criteria for GSE88884.
