# Supplementary material for: Integrative Bioinformatics Analysis Identifies DDX60 as a Potential Biomarker for Systemic Lupus Erythematosus
Source: Dis Markers. 2023 Jan 9;2023:8564650. doi: 10.1155/2023/8564650 (PMC9842429; doi:10.1155/2023/8564650)
Supplement: Supplementary Materials — Table S1: clinical information from GSE88884. Table S2: details of incomplete data of nine patients. Table S3: clinical information of 29 participants. Table S4: seven overlapped genes of DEGs and clinical feature-related genes from GSE88884. Table S5: simple logit regression of DDX60 expression in SLE/HC. Table S6: correlation analysis of DDX60 expression and SLEDAI in SLE patients. Information S1: patient inclusion criteria and exclusion criteria for GSE88884. [file 8564650.f1.docx]

Supplementary Materials

**Table S1** Clinical information from GSE88884

|  | N max min average [standard deviation](https://www.baidu.com/s?rsv_dl=selectedsearch&wd=standard%20deviation) |
| --- | --- |
| Age | 1751 87 18 41.52 12.174 |
| SLEDAI | 1751 40 2 10.38 3.762 |
| Anti-dsDNA | 1751 297 3 104.97 113.165 |

SLEDAI, Systemic lupus erythematosus disease activity index; anti-dsDNA, anti-double-stranded DNA;

**Table S2** Details of incomplete data of nine patients

| Probe name | Type of lacking data |
| --- | --- |
| GSM2351335 | SLEDAI, anti-dsDNA, C3, C4 |
| GSM2351570 | SLEDAI, anti-dsDNA, C3, C4 |
| GSM2351692 | C3, C4 |
| GSM2351775 | C3, C4 |
| GSM2351787 | anti-dsDNA |
| GSM2351903 | C3, C4 |
| GSM2352086 | SLEDAI, anti-dsDNA, C3, C4 |
| GSM2352170 | SLEDAI, anti-dsDNA, C3, C4 |
| GSM2352488 | C3, C4 |

SLEDAI, Systemic lupus erythematosus disease activity index; anti-dsDNA, anti-double-stranded DNA; C3, complement component C3; C4, complement component C4.

**Table S3** Clinical information of 29 participants

**SLE patients:**

**Health control:**

SLE, Systemic lupus erythematosus; HC, Healthy Control; SLEDAI, Systemic lupus erythematosus disease activity index; C3, complement C3 ; C4, complement C4 ; WBC, white blood cell ; NE , [neutrophil](https://cn.bing.com/dict/search?q=neutrophil&FORM=BDVSP6&cc=cn) [granulocyte](https://cn.bing.com/dict/search?q=granulocyte&FORM=BDVSP6&cc=cn) ; LY, [lymphocyte](https://cn.bing.com/dict/search?q=lymphocyte&FORM=BDVSP6&cc=cn) ; PLT, [platelet](https://cn.bing.com/dict/search?q=platelet&FORM=BDVSP6&cc=cn) ; HCQ, Hydroxychloroquine; MTX, methotrexate ; MMF, mycophenolate mofetil ; LEF, Leflunomide ; ESR, erythrocyte sedimentation rate; CRP, C-reactive protein; PRO, Protein; NIT, nitrite; GLU, glucosuria; LE, leukocyte esterase;  UEC, Urine epithelial cell; BIL, bilirubin; KET, Ketone; URO, urobilinogen;

**Table S4** Seven overlapped genes of DEGs and clinical features related genes from GSE88884

| Gene | *P*-values | | | | SigNum |
| --- | --- | --- | --- | --- | --- |
|  | anti-dsDNA | C3 | C4 | SLEDAI |  |
| DDX60 | 2.98e-37 | 2.96e-46 | 3.89e-25 | 4.57e-17 | 4 |
| IFI44L | 1.09e-36 | 4.22e-44 | 7.68e-25 | 4.58e-18 | 4 |
| IFI6 | 3.71e-38 | 5.38e-45 | 1.77e-25 | 2.00e-15 | 4 |
| IFI44 | 5.58e-35 | 2.51e-40 | 4.18e-21 | 1.61e-16 | 4 |
| RSAD2 | 1.79e-35 | 2.15e-40 | 4.93e-22 | 1.02e-14 | 4 |
| PLSCR1 | 1.30e-30 | 1.40e-35 | 4.01e-18 | 1.47e-13 | 4 |
| HERC5 | 9.07e-30 | 7.72e-34 | 1.70e-18 | 7.33e-12 | 4 |

anti-dsDNA, anti-double-stranded DNA; C3, complement component C3; C4, complement component C4; SLEDAI, Systemic lupus erythematosus disease activity index; SigNum, number of clinical assessments that have a *P*-value < 0.01.

**Table S5** Simple logit regression of DDX60 expression in SLE/HC

| Total  (N) | β1  (Best-fit values) | 95% CI for β1  (Profile likelihood) | Odds ratio | 95% CI for OR  (Profile likelihood) | *P*-value  (Wald test) | *P*-value  (Likelihood ratio test) |
| --- | --- | --- | --- | --- | --- | --- |
| 1810 | 1.528 | 1.202 to 1.903 | 4.608 | 3.328 to 6.706 | <0.0001 | <0.0001 |

CI: confidence interval; OR: odds ratio.

**Table S6** Correlation analysis of DDX60 expression and SLEDAI in SLE patients

| D'Agostino & Pearson test | | Spearman's test | | | |
| --- | --- | --- | --- | --- | --- |
| DDX60 | SLEDAI | Number of XY Pairs | r | 95% CI | P (two-tailed) |
| <0.0001 | <0.0001 | 1751 | 0.2352 | 0.1891 to 0.2803 | <0.0001 |

SLEDAI, Systemic lupus erythematosus disease activity index; r corresponds to the Spearman coefficient of correlation; CI: confidence interval.

**Information S1** Patient inclusion Criteria and exclusion criteria for GSE88884

Inclusion Criteria:

- Clinical diagnosis of SLE as defined by American College of Rheumatology (ACR) criteria
- Have positive antinuclear antibodies (ANA)
- Agree not to become pregnant throughout the course of the trial
- Have a screening SELENA-SLEDAI score ≥6. (The participant must be actively exhibiting all the symptoms scored on the screening SELENA-SLEDAI on the day of screening.)

Exclusion Criteria:

- Have active severe Lupus kidney disease
- Have active Central Nervous System or peripheral neurologic disease
- Have received intravenous immunoglobulin (IVIg) within 180 days of randomization
- Have active or recent infection within 30 days of screening
- Have had a serious infection within 90 days of randomization
- Have evidence or test positive for Hepatitis B
- Have Hepatitis C
- Are human immunodeficiency virus (HIV) positive
- Have evidence of active or latent tuberculosis (TB)
- Presence of significant laboratory abnormalities at screening
- Have had a malignancy in the past 5 years, except for cervical carcinoma in-situ or basal cell or squamous epithelial skin cell that were completely resected with no reoccurrence in the 3 yrs prior to randomization
- Have received greater than 40 mgs of prednisone or equivalent in the past 30 days
- Have changed your dose of antimalarial drug in the past 30 days
- Have changed your dose of immunosuppressive drug in the past 90 days
- Have previously received rituximab
